# Oxytocin and Sensory Network Plasticity

**DOI:** 10.3389/fnins.2020.00030

**Published:** 2020-01-29

**Authors:** Brandon T. Pekarek, Patrick J. Hunt, Benjamin R. Arenkiel

**Affiliations:** ^1^Genetics and Genomics Program, Baylor College of Medicine, Houston, TX, United States; ^2^Department of Molecular and Human Genetics, Baylor College of Medicine, Houston, TX, United States; ^3^Medical Scientist Training Program, Baylor College of Medicine, Houston, TX, United States; ^4^Department of Neuroscience, Baylor College of Medicine, Houston, TX, United States; ^5^Jan and Dan Duncan Neurological Research Institute, Texas Children’s Hospital, Houston, TX, United States

**Keywords:** oxytocin, plasticity, sensory, synapse, disease

## Abstract

An essential characteristic of nervous systems is their capacity to reshape functional connectivity in response to physiological and environmental cues. Endogenous signals, including neuropeptides, governs nervous system plasticity. Particularly, oxytocin has been recognized for its role in mediating activity-dependent circuit changes. These oxytocin-dependent changes occur at the synaptic level and consequently shape the cellular composition of circuits. Here we discuss recent advances that illustrate how oxytocin functions to reshape neural circuitry in response to environmental changes. Excitingly, recent findings pave the way for promising therapeutic applications of oxytocin to treat neurodevelopmental and neuropsychiatric diseases.

## Introduction

Our brains continually organize a multitude of sensory inputs into meaningful outputs. As humans, we subconsciously handle an ever-changing array of sensory information. Sensory inputs can range from the tantalizing smell of freshly prepared food, to the complexity of making an involved decision in a social hierarchy. Moreover, these inputs not only change rapidly over time but are often presented simultaneously. Human brains have evolved to accommodate rapid influxes of information through multiple forms of plasticity. Synaptic plasticity has long been accepted as the basis of learning and memory (Makino et al., 2016). However, within the past 30 years we have begun to appreciate that adult-born neurons have the capacity to continually integrate into existing circuitry in the adult mammalian brain to provide an additional means of plasticity (Alvarez-Buylla et al., 1990; Lois and Alvarez-Buylla, 1994). Both of these plasticity mechanisms work on two largely different timescales but serve the same purpose of adapting existing brain circuitry to benefit the organism.

Synaptic neuroplasticity is broadly defined as the property of synapses to strengthen or weaken in response to changes in presynaptic activity. This plasticity often manifests as a change to the number of synapses on a particular neuron, thereby altering brain-wide neural connectivity on a long-term scale. This process is guided by both environmental cues and gene expression within cells. Effectively, plasticity at both the synaptic and the cellular level allows for remodeling or rewiring of existing circuitry. Circuit remodeling can be guided by natural development or something more malevolent such as disease or injury. Interestingly, a number of genes associated with neuroplastic control are mutated in patients with psychiatric and autism spectrum disorders (ASDs). This connection suggests that brains that either cannot adapt appropriately to their environment or brains that adapt too quickly both manifest disease (Bourgeron, 2015). Therefore it is vital to understand neuroplastic mechanisms to treat these types of illnesses.

Almost exclusively, scientists have directed their attention toward small, fast-acting neurotransmitters when studying neuroplasticity and neurogenesis. This is largely due to their ability to transmit synaptic information quickly and efficiently in combination with their global presence in plastic brain regions. Decades of research dedicated to how fast neurotransmitters regulate synaptic plasticity has yielded great results, but somewhat at the expense of ignoring the contribution from neuropeptides. Recent research recognizes neuropeptides as key orchestrators of both initiating and maintaining plastic states within a diverse array of sensory systems. Neuropeptides are particularly interesting candidates because their release often correlates physiological stimuli, and brain state to changing circuit dynamics. Notably, work in the hippocampus links local neuropeptide release to environmental and social learning stressors or enrichment (Ögren et al., 2010; Grégoire et al., 2014; Li et al., 2017). Furthermore, neuropeptides have a long-lasting nature and broad effective areas, which make neuropeptides ideal brain state sensors and circuit regulators (Neumann, 2008; van den Pol, 2012).

Recently, the neuropeptide oxytocin has garnered interest as a potent modulator of both adult-born neurogenesis and synaptic plasticity (Zheng et al., 2014; Marlin and Froemke, 2017). Here, we describe a wide array of neuromodulatory functions that oxytocin serves in the context of synaptic plasticity and adult-born neuron integration. Though the behavioral effects of oxytocin are both species dependent and brain region specific, we focus this review on oxytocin-driven plasticity from experimental data in mice or rats. Additionally, we illustrate the consequences of oxytocin-dependent functions on the macroscopic scale of animal behavior. Finally, we reflect on this information and its therapeutic potential to intelligently rewire or replace diseased brain circuitry.

## Oxytocin at the Synapse

Oxytocin is a neurohypophysial ring nonapeptide produced centrally within the hypothalamus (Sofroniew, 1983). The processed form of the peptide is loaded into dense core vesicles and dispersed via axonal projections to distant brain regions. Oxytocin release may be synaptic or extrasynaptic, with broad-acting effects due to its long half-life and extensive release sites (Landgraf and Neumann, 2004; Ludwig and Leng, 2006). Supporting a predominant role for volume transmission of oxytocin, many studies have struggled to identify traditional peptide-expressing presynaptic and receptor-expressing postsynaptic partners. This conundrum has led the field to view oxytocin receptor expression as a key mode of regulation within the oxytocin system.

**FIGURE 1:**
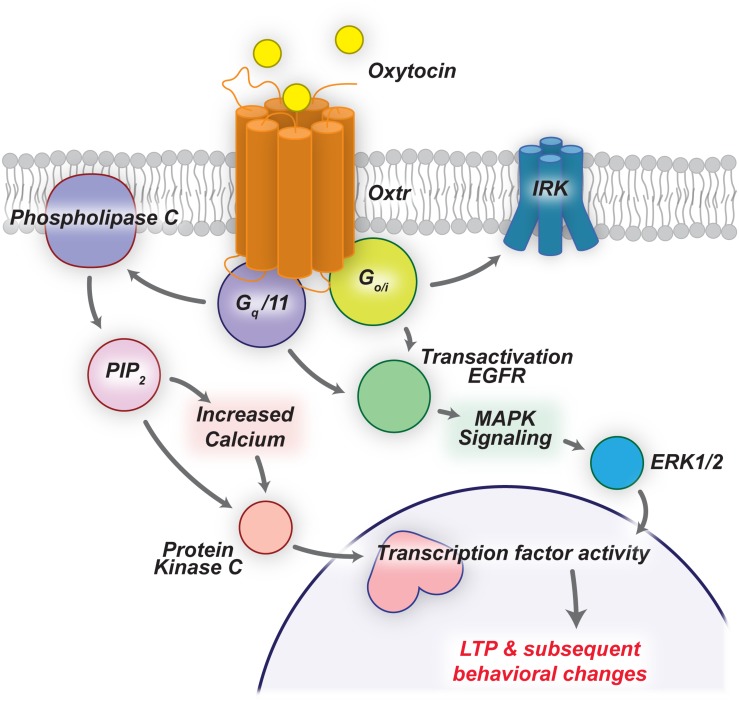
Oxytocin signaling cascades lead to LTP and behavioral changes. OXTRs are G-protein-coupled-receptors that can activate a number of downstream pathways upon ligand binding. Here we highlight two such pathways that drive LTP in the mammalian brain.

Oxytocin Receptor (OXTR) is a 7-transmembrane domain G-protein-coupled-receptor that is broadly expressed throughout the mammalian brain (Figure 1; Gimpl and Fahrenholz, 2001; Busnelli and Chini, 2018). In neurons, OXTR can couple to both G_q_ and G_o_/G_i_ protein subunits, leading to variable phosphorylation of PLCβ and PKC, activation of EGFR and MAPK cascade, or activation of inward rectifying potassium channels (Busnelli and Chini, 2018). Interestingly, Busnelli and Chini (2018) show that coupling of different G proteins, and ultimately the intracellular cascade of events caused by OXTR activation, depends on the expression levels of OXTR, the concentration of oxytocin present, and the expression of specific G protein subunits. Therefore, due to the diverse repertoire of *OXTR* associated signaling cascades, OXTR activation has a wide variety of downstream effects, which can readily be seen by comparing differential *Oxtr* and G protein subunit expression.

**FIGURE 2:**
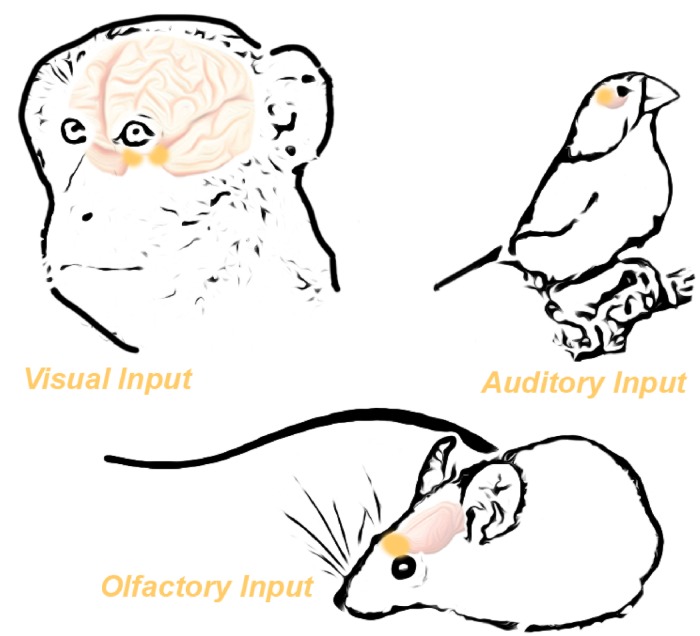
Oxytocin receptor expression in the brain is functionally specific across species. High *Oxtr* expression in the visual pathways of primates correlates with the heavy reliance of primates on the visual system. Similarly, birds demonstrate high *Oxtr* expression in the auditory system, mirroring their heavy reliance on auditory signaling. Finally, mice potently express *Oxtr* in the olfactory bulb, reflecting their reliance on olfactory cues. Adapted from Grinevich et al. (2016).

Expression of *Oxtr* in distinct brain regions facilitates species-specific behaviors (Gimpl and Fahrenholz, 2001; Grinevich et al., 2016). One notable illustration of this is the monogamous prairie vole, in which well-defined expression of *Oxtr* in only a few distinct brain regions drives the development of monogamous behavior in prairie voles; however, lack of *Oxtr* expression in meadow voles yields a more solitary animal (Young and Wang, 2004). Indeed, we can see that *Oxtr* expression is enriched in sensory systems important for social behavior on a species-by-species basis (Figure 2). For example, animals that primarily communicate via visual cues have an enrichment of OXTR in visual-responsive brain regions (Figure 2). The correlation between regional expression of *Oxtr* and species-specific behavior suggests a common evolutionary function of oxytocin. Specifically, oxytocin has the capability to induce or re-open a critical period within existing brain circuitry in sensory systems (Nardou et al., 2019). The consequence of this can be seen both molecularly at the synapse, and also as modified behavioral outputs.

Oxytocin’s most famous role in popular science culture is as “the love hormone” due to the trust and positive feelings Oxytocin engenders toward others (Magon and Kalra, 2011). For example, Oxytocin effects rodent maternal behavior by inducing potent and long-lasting synaptic changes to drive and maintain this nurturing behavior. Recently Marlin et al. (2015) demonstrated that *Oxtr* enrichment in a small domain of the mouse auditory cortex (A1) sensitizes mothers to newborn pup ultrasonic vocalizations; endogenous oxytocin release and subsequent OXTR activation in A1 induces both temporal and spatial gating of A1 activity in the maternal mice in response to their newborn pup calls. Additionally they showed that oxytocin, when paired with pup calls, triggers a disinhibitory effect (Marlin et al., 2015). Importantly, this group later demonstrated that this particular oxytocin signaling mechanism is mediated largely by NMDA-dependent long-term potentiation (LTP) (Mitre et al., 2016), whereby oxytocin promotes a disinhibitory effect that facilitates spiking of normally sub-threshold responses, which is then further strengthened and consolidated by NMDA-dependent LTP. Taken as a whole, oxytocin sensitizes maternal mice to the sounds of their pup calls by increasing the signal-to-noise ratio via disinhibition in the auditory cortex. This drives a precise, unambiguous and meaningful response by A1 neurons of the mother to her own pups, establishing a basis for maternal behavior.

In other sensory cortices, oxytocin increases meaningful excitation, thus driving canonical synaptic plasticity. Sensory-deprived animals demonstrate reduced oxytocin synthesis in the hypothalamus and decreased transmission of oxytocin to the corresponding sensory cortex (ex. S1 in whisker-deprived mice). This reduction in synthesis and overall transmission results in lower excitatory activity within the sensory cortex. However, upon excitation of oxytocin-carrying fibers, or *in vivo* oxytocin application, increased excitatory activity rescues the synaptic effects of sensory deprivation. This effect correlates strongly with cortical synaptic plasticity that manifests from environmental enrichment, which also drives increased oxytocin levels. Thus, oxytocin mediates cortical synaptic plasticity that accompanies sensory enrichment (Zheng et al., 2014). These findings further corroborate the view that oxytocin drives plasticity by enhancing the signal-to-noise ratio throughout various brain regions.

In a similar fashion, but via a different mechanism, oxytocin increases the signal-to-noise ratio of rat socio-sexual olfactory cues. The main olfactory bulb (MOB) inhibitory interneurons, or granule cells, laterally inhibit the principle MOB excitatory neurons to filter olfactory signaling prior to downstream cortical processing. Oettl et al. (2016) demonstrated that oxytocin recruits top-down cortical inputs to drive activation of the MOB granule cells. This sensory modulation drives a greater representation of rat social odorants, facilitating better social memory and recognition (Oettl et al., 2016). In this respect, olfaction implements oxytocin-mediated circuit level plasticity to drive enhanced social salience.

Recent research indicates that Oxytocin directly drives the formation of stronger synapses, thereby improving the signal-to-noise ratios of regions that mediate memory-related behaviors. Hippocampal synaptic malformation from region- and cell-specific deletion of *Oxtr* results in impaired long-term social recognition memory development (Raam et al., 2017). Conditional loss of oxytocin signaling has no effect on sociability, preference for social novelty, or anxiety-related behaviors. Importantly, neurons with *Oxtr* deletions exhibit defects in LTP induction; however, there are no defects in long-term depression (LTD). *Ex vivo*, OXTR blockers are sufficient to potentiate excitatory synaptic responses that rely on NMDA receptor activation (Lin et al., 2018). Additionally, oxytocin application drives dendritic and synaptic refinement in cultured glutamatergic hippocampal neurons (Ripamonti et al., 2017), which supports a model in which oxytocin drives fewer but stronger synapses. Together, these findings support that oxytocin plays an important role in enhancing excitatory connections within the hippocampus to mediate LTP and social memory formation while also mediating aspects of adult-born neurogenesis in the hippocampus. Thus, in conjunction with mediating mechanisms of synaptic plasticity in developing neurons, oxytocin potently regulates neurogenic plasticity as well.

## Oxytocin and Adult Neurogenesis

**FIGURE 3:**
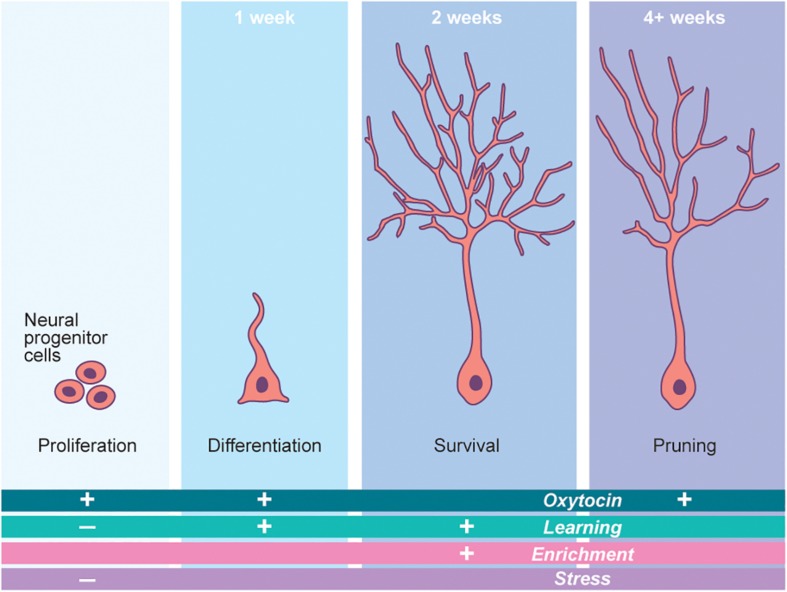
Neurogenesis is a potent driver of neural plasticity in the brain. Adult born neurogenesis follows a stereotyped developmental pathway. Each step of this pathway is modified by a number of outside factors. Oxytocin potently regulates this process in a number of ways, thus governing cellular plasticity in the brain. The time periods marking the top of each developmental step refer to the time at which these steps occur in mice. The “+” and “−” signs denote the increase or decrease, respectively, of each step (Proliferation, etc.) in the presence of the factor (Oxytocin, etc.) associated with each sign. Adapted from Gage (2019).

There are two well-established niches for adult neurogenesis in the mammalian brain: the dentate gyrus (DG) of the hippocampus and the subventricular zone (SVZ), which supplies new neurons into the olfactory system. Adult neurogenesis in the hippocampus begins in the DG with new-neurons which undergo stereotyped developmental programs to become mature dentate granule cells (Toda et al., 2019). Development and integration of adult-born neurons is largely dependent on the physiological state of the animal, with immature neurons displaying a bona fide activity-dependent critical period at roughly 3 weeks after new neurons are born (Figure 3; Ge et al., 2007). Continuous adult neurogenesis in the hippocampus may provide both the structural and functional plasticity necessary for complex behaviors such as learning and memory formation (Kempermann et al., 2004). Although the phenomenon of adult neurogenesis itself is well studied in the hippocampus, the field is only beginning to unravel the presynaptic cues that regulate this process. The discovery of signaling molecules linking an animal’s physiological state with the activity or circuit integration status of newly born neurons is critically important.

Understanding oxytocin-mediated hippocampal plasticity is relatively nascent; however, recent seminal discoveries implicate neuropeptide hormones as potential candidates to bridge the gap between physiological state and newborn neuron integration. Namely, oxytocin encompasses dual roles in both regulating adult-neurogenesis and driving adult-born neuron circuit integration. *Oxtr* is highly expressed in developing neurons in both the CA2 and CA3 regions of the hippocampus (Lin et al., 2018). Additionally, long range oxytocin-containing varicosities extend from the hypothalamus to the hippocampal neurogenic niche, thereby providing a source of oxytocin to newborn neurons (Landgraf and Neumann, 2004). The importance of oxytocin signaling in newborn neurons is highlighted by experiments that genetically delete *Oxtr* from the hippocampus to effectively and dramatically reduce newly generated neurons (Lin et al., 2017). Conversely, exogenous application of oxytocin to the hippocampus increases neuronal proliferation in rats (Sánchez-Vidaña et al., 2016). However, these effects are thought to be non-cell autonomous, as oxytocin increases the excitability of CA3 neurons, whose activity directly regulates hippocampal adult-neurogenesis. Interestingly, when *Oxtr* was removed from newborn hippocampal granule cells during the developmental critical period, marked reduction in dendritic complexity and delays in the excitatory-to-inhibitory GABA switch were observed (Lin et al., 2017). Therefore, oxytocin alters the neuronal activity of surrounding newborn neurons, thereby shaping their dendritic morphologies and firing properties. This concept is further illustrated by experiments in which administration of exogenous oxytocin increases the differentiation and complexity of newborn neuron dendrites (Sánchez-Vidaña et al., 2016).

Oxytocin-mediated cellular changes ultimately alter hippocampal activity, thereby driving behavioral phenotypes. This behavioral change is best illustrated by the attenuation of novel social interactions in mice that have hippocampal deleted *Oxtr*. Such attenuation implies a social memory formation defect (Raam et al., 2017; Lin et al., 2018). Thus, oxytocin-mediated development in newborn neurons may ultimately govern how animals respond to and integrate critical social stimuli.

## Oxytocin and Adult Neurogenesis in the Olfactory System

Throughout the rodent brain, regions densely innervated by oxytocin-releasing fibers also strongly express *Oxtr* (Knobloch et al., 2012). This robust overlap indicates functional connectivity and potential oxytocin signaling programs in these brain areas (Grinevich et al., 2016). However, one of the few spots that exhibit ligand-receptor mismatch is the MOB. Notably, the mammalian olfactory system constitutes one of the most potent neurogenic niches, receiving tens of thousands of new adult-born granule cells (abGCs) every day (Alvarez-Buylla and García-Verdugo, 2002; Petreanu and Alvarez-Buylla, 2002). These cells begin as progenitors in the SVZ, migrate through the rostral migratory stream exposed to a milieu of extrasynaptic neurotrophic cues, and arrive in the MOB where they undergo precise developmental programs and integrate into existing olfactory circuitry (Carleton et al., 2003). Similar to embryonic neuronal development, abGCs rely on presynaptic activity for survival during their developmental critical period (Yamaguchi and Mori, 2005; Kelsch et al., 2009). Neuropeptides are attractive candidates to convey physiological and/or olfactory activity to incoming abGCs. In fact, abGCs express a number of neuropeptide receptors (Gould and Zingg, 2003; Lein et al., 2007; Garcia et al., 2014). We have found that local neuropeptidergic activity from corticotropin releasing hormone (CRH) producing cells drive synaptic development and survival throughout this critical period. Genetic ablation of CRH signaling in abGCs causes cell death and malformed synapses, while over activation of the CRH receptor (CRHR) in abGCs mediates increased survival and synaptic integration (Garcia et al., 2014). MOB abGCs express moderate to high levels of *Oxtr*, but do not receive direct input from any identifiable oxytocin-releasing projections (Grinevich et al., 2016). The etiology and consequences of this mismatch are not well understood. However, the strong expression of *Oxtr* in developing adult-born neurons offers the potential for functional relevance within the scope of adult-born neuron development, synapse formation, and circuit integration.

Intriguingly, granule cells throughout the entire MOB are highly plastic and exhibit a high turnover of dendritic spines throughout their development (Sailor et al., 2016). This structural plasticity at the synaptic level is thought to underlie the overall flexibility and plasticity of mouse olfactory circuitry. Oxytocin’s well-known role in mediating plasticity in other areas of the brain, combined with the high expression of *Oxtr* in the MOB, make oxytocin an intriguing candidate of study. Indeed, early work indicates that local application of oxytocin directly to the MOB enhances the activity of local inhibitory granule cells (Oettl et al., 2016), supporting the notion that OXTR is functional in these neurons; however, it is unclear if oxytocin plays an important role in mediating adult-born neuron plasticity in the MOB.

## Behavioral and Disease States Affected by Oxytocin

Oxytocin is known for its role in mediating neural circuits that govern complex mammalian behaviors. Many of these behaviors involve social interactions and rely on social-based learning. Similarly, defective oxytocin-related circuitry can result in human diseases that include social deficits such as Autism Spectrum Disorders (ASDs), which are characterized by deficits in communication and social interactions (Feldman et al., 2016; Guastella and Hickie, 2016; Neumann and Slattery, 2016). The link between oxytocin and ASDs is substantiated by findings that both polymorphisms in *Oxtr* and methylation changes in the *Oxtr* promoter are associated with ASD diagnoses (LoParo and Waldman, 2015; Elagoz Yuksel et al., 2016). Moreover, treating ASD patients with oxytocin has led to symptom improvement (Hollander et al., 2007; Andari et al., 2010; Striepens et al., 2011).

Importantly, mouse models in which oxytocin signaling has been disrupted recapitulate many of the symptoms seen in ASD patients (Lee et al., 2008; Sala et al., 2011). These findings imply that oxytocin signaling potently affects mammalian behavior and offers the field a powerful rodent model to develop therapies for these behavioral disorders. Mouse models of oxytocin signaling may also lead toward a better understanding of other syndromic behavioral disorders. For example, models of fragile-X syndrome show impairments in developmental inhibitory-to-excitatory switch of young neurons regulated by oxytocin signaling, implicating disruptions in the oxytocin pathway (Tyzio et al., 2006). Other models include aberrant dendritic phenotypes seen in *Fmrp* knockout models of fragile-X syndrome, along with other rodent models of ASDs (Galvez et al., 2003; Kulkarni and Firestein, 2012). These data not only supports previous experiments regarding the effects of oxytocin on neurons throughout several brain regions, but it also supports data in which ASD patients manifest aberrant dendritic morphology during development (Bennett and Lagopoulos, 2015; Lin et al., 2017).

Such morphological changes, combined with alterations in LTP downstream of oxytocin signaling, identify oxytocin as a potential mediator of excitatory-inhibitory balance in human neural circuits (Ripamonti et al., 2017). Imbalances between excitation and inhibition have long been associated with neuropsychiatric disorders, including ASD (Selten et al., 2018). Interestingly, a growing body of literature highlights that many of the genes implicated in ASDs act within or around the synapse, further supporting that excitation-inhibition balance is critical in ASD etiology. Furthermore, these findings also suggest oxytocin signaling as a potential therapeutic target for these diseases (Kleijer et al., 2014).

Finally, though much attention has been paid to the link between oxytocin signaling and ASDs, a number of other neuropsychiatric illnesses also show provocative links to oxytocin signaling (Kawamura et al., 2010; Rubin et al., 2014, 2018; Kirsch, 2015). Thus, discovering the neural and circuit-relevant mechanisms of oxytocin signaling will yield important information regarding the etiology of these diseases. Importantly, such efforts will also pave a path toward developing therapies for neuropsychiatric illnesses, and perhaps reshape the way we think about both disease states and patients who suffer from them.

## Conclusion

We are only beginning to appreciate neuropeptide modulation of plastic brain circuits. From social reward signaling to modulating olfactory representations of kin scents, neuropeptides are essential to relay environmental stimuli that guide changes in brain circuitry. Neuropeptides are one of many factors that contribute to activity-dependent changes in the brain, and oxytocin in particular is a potent regulator of both synaptic and cellular plasticity.

Oxytocin operates through both synaptic and cellular plasticity mechanisms to rewire brain circuitry to increase neuronal representation of sensory stimuli. This increased sensory salience facilitates both the formation and maintenance of complex behaviors. Disruptions in this system, particularly during sensitive critical developmental periods, may cause behavioral malformations due to underlying circuit imbalances.

The information we gain from rodent-based studies of oxytocin and neuropeptide signaling will allow us to continue to dissect the complexities of neural plasticity, which sit at the heart of many neuropsychiatric and behavioral disorders. Advances in our understanding of neuropeptide signaling provide insight into the mechanisms of neuroplasticity while also offering hope for future treatments of neuropsychiatric illnesses. Excitingly, by understanding oxytocin’s influence on plasticity, investigators have begun to co-opt the oxytocin system to treat developmental behavioral disorders (Guastella et al., 2010; Yatawara et al., 2016; Parker et al., 2017). The advent of new methods to better diagnose and understand behavioral disorders will allow us to appreciate neural plasticity as a common underlying mechanism, and provide needed insight toward treating these disorders by leveraging neuropeptide biology.

## Author Contributions

BP and PH wrote the first draft of the manuscript. BA supplied their technical knowledge to support the manuscript throughout the revision process. PH created the figures for the manuscript. All authors contributed to the manuscript revision and approved the submitted version.

## Conflict of Interest

The authors declare that the research was conducted in the absence of any commercial or financial relationships that could be construed as a potential conflict of interest.
